# *t10c12* Conjugated Linoleic Acid Suppresses HER2 Protein and Enhances Apoptosis in SKBr3 Breast Cancer Cells: Possible Role of COX2

**DOI:** 10.1371/journal.pone.0005342

**Published:** 2009-04-28

**Authors:** Margaret Flowers, Patricia A. Thompson

**Affiliations:** 1 Department of Nutritional Sciences, University of Arizona, Tucson, Arizona, United States of America; 2 Department of Epidemiology, Mel and Enid Zuckerman College of Public Health, Arizona Cancer Center, University of Arizona, Tucson, Arizona, United States of America; Cleveland Clinic, United States of America

## Abstract

**Background:**

HER2-targeted therapy with the monoclonal antibody trastuzumab (Herceptin®) has improved disease-free survival for women diagnosed with HER2-positive breast cancers; however, treatment resistance and disease progression are not uncommon. Current data suggest that resistance to treatment in HER2 cancers may be a consequence of NF-κB overexpression and increased COX2-derived prostaglandin E2 (PGE_2_). Conjugated linoleic acid (CLA) has been shown to have anti-tumor properties and to inhibit NF-κB activity and COX2.

**Methods:**

In this study, HER2-overexpressing SKBr3 breast cancer cells were treated with *t10c12* CLA. Protein expression of the HER2 receptor, nuclear NF-κB p65, and total and phosphorylated IκB were examined by western blot and immunofluorescence. PGE_2_ levels were determined by ELISA. Proliferation was measured by metabolism of 3-(4, 5-Dimethylthiazol-2-yl)-2, 5-diphenyltetrazolium bromide (MTT), and apoptosis was measured by FITC-conjugated Annexin V staining and flow cytometry.

**Results/Conclusions:**

We observed a significant decrease in HER2 protein expression on western blot following treatment with 40 and 80 µM *t10c12* CLA (p<0.01 and 0.001, respectively) and loss of HER2 protein in cells using immunoflourescence that was most pronounced at 80 µM. Protein levels of nuclear NF-κB p65 were also significantly reduced at the 80 µM dose. This was accompanied by a significant decrease in PGE_2_ levels (p = 0.05). Pretreatment with *t10c12* CLA significantly enhanced TNFα-induced apoptosis and the anti-proliferative action of trastuzumab (p = 0.05 and 0.001, respectively). These data add to previous reports of an anti-tumor effect of *t10c12* CLA and suggest an effect on the HER2 oncogene that may be through CLA mediated downregulation of COX2-derived PGE_2_.

## Introduction

Overexpression of the HER2 oncogene occurs in 25–30% of human breast cancers and is associated with poor outcome [1]. HER2 overexpression often occurs with estrogen receptor (ER) negative disease, making these tumors resistant to hormonal therapies [2]. Treatment with trastuzumab (Herceptin®) has improved disease-free survival in patients with metastatic breast cancer, but is limited by both cardio toxicity and inherent and acquired resistance [3]. Significant effort is currently directed at combining Herceptin® with traditional anticancer agents as well as emerging therapies against additional target molecules, including inhibitors of other receptor tyrosine kinases, nuclear factor-κB (NF-κB), and chaperone protein HSP90 to improve clinical outcome [2], [4], [5], [6].

One rationale for the use of combination therapies is to modulate multiple, deregulated tumor targets to reduce the likelihood of acquired resistance to the primary therapy. The molecular basis for acquired resistance to Herceptin® is poorly understood, but may involve HER2-independent upregulation of phosphoinositide 3 (PI3) and mitogen activated protein (MAP) kinase pathways, possibly through upregulation of insulin-like growth factor-1 receptor (IGF-IR) or EGFR ligand activation [7]. Chemotherapy-induced NF-κB expression attenuates the intended cell killing effect and may play a role in drug resistance that is often seen in HER2 and EGFR overexpression [8], [9].

NF-κB is a key transcription factor in the regulation of the inflammatory response [10]. In basal conditions, NF-κB is sequestered in the cytoplasm by the inhibitor- κB (IκB) complex [11]. Activation occurs when the inhibitor of IkB, IκB-kinase (IKK) phosphorylates IκB, releasing NF-κB to migrate to the nucleus and regulate the expression of genes involved in tumor promotion and progression such as growth factors, cell cycle regulators, anti-apoptotic proteins, stromal remodeling proteases, angiogenic factors, and cell adhesion molecules [9], [11], [12], [13]. Constitutive activity of NF-κB has been reported in a number of cancers [14], [15], [16], [17], [18], [19], [20] and is known to inhibit apoptosis and promote tumorigenesis through regulation of proliferation, angiogenesis, invasion, and metastasis [21], [22], [23], [24]. In breast cancer, constitutive activity of NF-κB causes loss of estrogen receptor (ER) and resistance to chemo-, radiation-, and antibody-based therapies through signaling events downstream of ERBB2 or EGFR [9], [19], [25]. One of the key targets of NF-κB is the inducible cyclooxygenase COX2, the enzyme responsible for the conversion of arachadonic acid to prostaglandin (PG)E_2_ [26]. COX2 and PGE_2_ have been implicated in the progression of breast and other cancers and may act to sustain HER2 signaling [27], [28], [29].

Conjugated linoleic acid (CLA) belongs to a group of conjugated diene isomers of linoleic acid that are predominantly produced in the rumen of ruminant animals and available as dietary supplements for weight loss. The two most prominent isomers, *cis*9-*trans*11 (*c9t11*) and *trans*10-*cis*12 (*t10c12*), have been shown to have anti-tumor activity including proapoptotic activity [30], [31], inhibitory effects on cell cycle progression, proliferation [32], [33], and angiogenesis [34]. NF-κB-induced signaling is a frequent consequence of the upregulation of PI3 and MAP kinase pathways in cancer [11] and has been demonstrated to be a target of CLA in both cancer and non-cancer cells [35], [36], [37].

Though CLA has been shown to inhibit HER2 and ERBB3 protein expression in a colon cancer model [38], [39], its effect in a HER2 breast cancer model has not been previously investigated. Based on the reported associations between NF-κB and HER2 overexpression in breast cancer, we investigated the effect of CLA on cell growth characteristics in the HER2 overexpressing SKBr3 breast cancer cells with the hypothesis that CLA would inhibit HER2 by downreguating NF-κB signaling. Our results support an isomer-specific inhibitory effect of *t10c12* CLA on HER2 protein expression and membrane association in SKBr3 breast cancer cells. Our data provide evidence that this effect may be a consequence of CLA-induced downregulation of COX2-induced PGE_2_ production secondary to inhibition of NF-κB activity.

## Materials and Methods

### Reagents

Trypsin-EDTA, RPMI, PBS, fatty acid-free BSA, and DMSO were obtained from CellGrow (Herndon, VA). Fetal bovine serum was obtained from Atlas Biologicals (Fort Collins, CO). Penicillin/streptomycin was obtained from GIBCO/Invitrogen (Carlsbad, CA). Anti-beta actin, Annexin V-FITC Apoptosis Detection Kit (APOAF), C75, HEPES buffer, DTT, MgCl_2_, NaCl, and KCL were obtained from Sigma (St. Louis, MO). TritonX-100 was obtained from Pharmacia Biosciences (Piscataway, NJ). Tissue culture hardware was obtained form Nalge Nunc (Rochester, NY). CLA isomers *cis*9-*trans*11 (*c9t11*) and *trans*10-*cis*12 (*t10c12*) were obtained from Matreya (Pleasant Gap, PA. CLA was complexed to FA-free BSA in a 4∶1 molar ratio with 1 mM BSA stock. Antibodies against HER2 were obtained from Abcam (Cambridge, MA) for Western blot and from Calbiochem/Oncogene (Gibbstown, NJ) for immunofluorescence. Antibodies against NF-κB p65, IκB-α, phospho-IκB-α (Ser32), and p53 were obtained from Cell Signaling (Danvers, MA), as were the positive control lysates (#9243) for Western blot of NF-κB related proteins. Fluorescent conjugated anti-mouse (Alexa Fluor 488) and anti-rabbit (Alexa Fluor 564) were obtained from Molecular Probes/Invitrogen (Carlsbad, CA). ELISA EIA kit for PGE_2_ determination was obtained from Cayman Chemical (Ann Arbor, MI). The MTT viability assay was obtained from Roche Applied Science (Indianapolis, IN). Herceptin® (Genentech, San Francisco, CA) was generously supplied by the Arizona Cancer Center Clinic in a stock solution of 21 mg/ml.

### Cell Culture

The *Her2*-overexpressing SKBr3 were obtained from American Type Culture Collection (Manassas, VA) and independently authenticated. Cells were cultivated in McCoy's 5A media supplemented with 1.5 mM L-glutamine (Hyclone, Logan, UT), 10% FBS and 1% penicillin/streptomycin, and maintained at 37°C and 5% CO_2_. Cells utilized in these experiments were confirmed to be mycoplasma free. Unless otherwise indicated, all treatments were performed in growth media.

### Cell Viability/Proliferation/Apoptosis

Cell viability was determined by trypan blue exclusion using Beckman Coulter Counter. Proliferation was determined by metabolic activity and the reduction of MTT (3-(4,5-Dimethylthiazol-2-yl)-2,5-diphenyltetrazolium bromide, a tetrazole). For proliferation assays, 2.5×10^4^ cells/well/100 µl media were seeded in 96-well plates, allowed to adhere overnight, then washed and treated with *t10c12* CLA or vehicle as specified in figure legends. Determination of metabolic reduction of MTT was performed according to manufacturer's protocol (Roche Diagnostics, Mannheim, Germany). Absorbance was measured at 580 and 690 nm, using a microplate reader (Biotek Synergy 2) in accordance with the manufacturer's protocol. Apoptosis was measured by FITC conjugated Annexin V staining according to manufacturer's protocol (Sigma APOAF). Absorbance was read by flow cytometry using FACScan (BD Biosciences, San Jose, CA) in accordance with the manufacturer's protocol.

### Preparation of Whole-Cell Lysates

Cell pellets were washed two times in cold PBS and centrifuged at 2000 rpm for 10 minutes, then resuspended in ice-cold RIPA buffer (1× PBS, 1% NP-40 (nonidet P40, Sigma) 0.1% SDS) containing 1 mM phosphatase inhibitor, sodium orthovanadate, and HALT protease inhibitor cocktail (Pierce/Thermo Scientific, Rockford, IL). Protein concentration was determined by Pierce Micro BCA.

### Preparation of Nuclear Extracts

Cells were washed and harvested by trypsinisation, centrifuged to remove media, and washed twice in cold PBS. The protocol for nuclear extraction has been previously described [40]. All steps were performed at 4°C. Briefly, cell pellets were resuspended in hypotonic lysis buffer (10 mM HEPES pH 7.9, 1.5 mM MgCl_2_, 10 mM KCL, 0.5 mM fresh DTT, 1× HALT protease inhibitor cocktail, and 0.1% TritonX-100) and transferred to microcentrifuge tubes. Tubes were vortexed at 16,000 rpm for 15 seconds and allowed to incubate one hour at 4°C on a rocking platform. Tubes were centrifuged at 16,000 rpm for 15 minutes, and the supernatant containing the cytoplasmic extract was removed. Nuclear pellets were resuspended in 10 µl/∼8–10×10^6^cells nuclear extract buffer (20 mM HEPES pH7.9, 25% glycerol, 420 mM NaCl, 1.5 mM MgCl_2,_ 0.2 mM EDTA, 0.5 mM fresh DTT, and 1× HALT.) Samples were vortexed at the highest setting for 15 seconds and incubated on ice for 30 minutes, vortexing every 10 minutes. Tubes were centrifuges as before. Supernatant containing nuclear extracts were diluted 1∶4 in storage buffer (20 mM HEPES pH7.9, 20% glycerol, 1.5 mM MgCl_2,_ 100 mM KCL, 0.2 mM EDTA, 0.5 mM fresh DTT, and 1× HALT.) Protein concentration was determined by Bio-Rad Assay (500-0006). Extracts were stored at −80°C until use.

### Immunoblot

10–25 µg protein was loaded into 8 or 10% gels and separated by SDS PAGE using the Bio-Rad Criterion Gel system (Hercules, CA). Proteins were transferred to polyvinylidene difluoride membrane (Amersham Biosciences, Piscataway, NJ) by electroelution. Membranes were immunoblotted using standard protocols. Pierce Super Signal Dura West Substrate™ was used for visualization of protein bands. For successive antibody probes, blots were stripped using Pierce Restore™ stripping reagent with modifications to the manufacturer's protocol. Removal of both primary and secondary antibodies was confirmed by ECL detection before reprobing. Densitometry of bands was performed using Scion Image version Alpha 4.0.3.2. Protein levels were normalized to beta actin or total protein and reported relative to vehicle (as described in figure legends).

### Immunofluorescence

Cells were grown on 12-mm glass coverslips in six-well plates at a seeding density of 4×10^5^ cells/well/3 ml. After overnight adherence, cells were treated with 40 or 80 µM *t10c12* CLA or BSA for 24 hours. Cells were washed briefly in 1× PBS, 0.1% NaN_3_, before fixation in 2% paraformaldehyde (Pierce) for 20 minutes, and were then permeabilized in 0.2% Tween 20 (Sigma) for 5 minutes. Coverslips were blocked in 10% goat serum for one hour at room temperature and incubated overnight with antibodies prepared in blocking buffer (1×PBS, 0.1% NaN_3_, 10% goat serum, 1% TritonX). After incubation of primary antibodies, coverslips were washed 3×5 minutes in washing buffer (1× PBS, 0.1% NaN_3_, 1% Tween 20). Dilutions of Alexa Fluor 488 anti-mouse or 564 anti-rabbit were prepared in blocking buffer at 1∶600 and 1∶1000, respectively. Coverslips were incubated with secondary antibodies for one hour at room temperature. Coverslips were washed as before, then fixed in 100% ethanol before being applied to 3×1 mm microscope slides (Fisher Scientific, Pittsburgh, PA ) using Dako (Glostrup, Denmark) fluorescent mounting medium. Images were taken at 40× with Applied Precision (Issaquah, Washington) Delta Vision deconvolution microscope with SoftwoRX 3.5.0 software. Negative controls were incubated without primary antibody.

### Prostaglandin E2 (PGE_2_) Determination

Cells were grown to confluence, harvested by trypsinisation, and seeded in six-well plates at a seeding density of 1.0×10^6^ cells/well/3 ml. After adherence overnight, cells were treated with *t10c12* CLA at the indicated concentrations and time points. One hour before harvesting, media was removed and replaced with serum-free media containing 15 µM arachadonic acid as substrate for COX2. Supernatant was collected after treatment and centrifuged to remove cellular debris. An aliquot from each treatment was stored at −80°C until use. Supernatant was prepared in three dilutions using culture media (1∶10, 1∶20, and 1∶40), and PGE_2_ concentration was determined by PGE_2_ ELISA-based immunoassay (Cayman Chemical, Ann Arbor, MI). Negative controls did not receive arachadonic acid. The cells from these treatments were harvested and counted, and viability was determined by trypan blue. Cell pellets were then prepared for immunoblot.

### Statistics

Statistically significant differences between treatments were detected using the Student T test with a significance level of 0.05.

## Results

### The *t10c12* CLA Isomer Inhibits HER2 Expression in SKBr3

**Figure 1:**
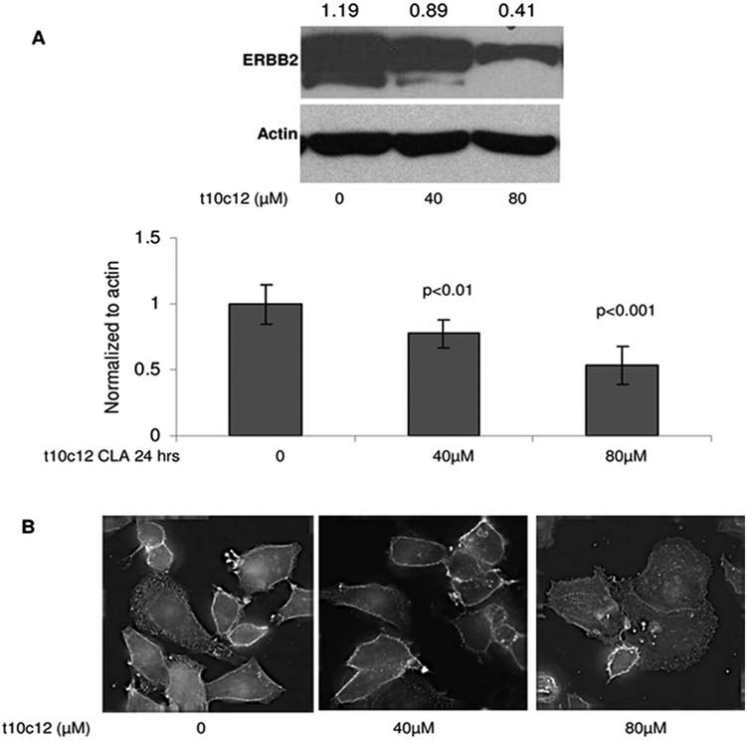
*t10c12* CLA reduces HER2 protein in SK-Br3 cells. (A) Representative western blot of total HER2 protein in response to 24 hr treatment with *t10c12* CLA. Cells were plated in 6-well plates, 3 wells per treatment. Cells from 3 wells were pulled and total protein was extracted as described in Materials and Methods. 25 µg of whole cell lysate were loaded into 8% gels. Densitometry of bands was performed using Scion Image software Alpha 4.0.3.2. Expression of HER2 was normalized to beta actin and compared to vehicle treatment. Values represent the mean +/− std error relative to vehicle from 5 independent experiments. (B) Immunofluoresnce of HER2 protein in SK-Br3 cells following 24 hr treatment with 40 µM (middle panel), 80 µM (right panel) or vehicle (left panel). Cells were treated and immunofluorescence was performed as described in Materials and Methods. Images were obtained using Delta Vision deconvolution microscope with SoftwoRX 3.5.0 software at 40× and optimized using Adobe PhotoShop CS2 version 9.0.2.

The SKBr3 breast cancer cell line overexpresses the HER2 protein due to genomic amplification at chromosome 17q12 [41]. Figure 1 shows that *t10c12* CLA significantly inhibited HER2 protein expression in SKBr3 cells by Western blot analysis in a dose-dependent manner (Figure 1A). This effect was not seen with the *c9t11* isomer at the concentrations and time points examined (data not shown). In agreement with Western blots, immunofluorescence staining of cells treated with *t10c12* CLA indicated a reduction in surface and membrane HER2 protein at both 40 and 80 µM treatment (Figure 1B). 

**Figure 2:**
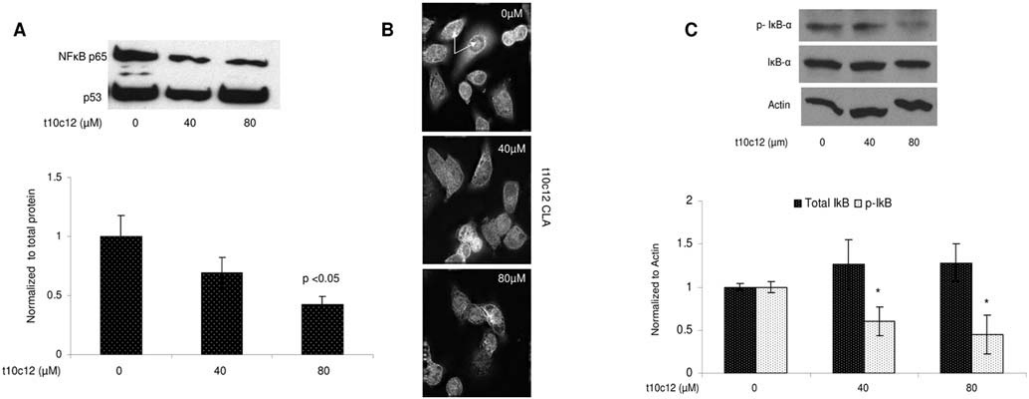
*t10c12* CLA reduces nuclear p65 in SKBr3 cells. (A) Western blots of NFκB p65 in nuclear extracts. Cells were plated in 6-well plates, 3 wells per treatment. Cells from 3 wells were pulled and nuclear extract was obtained as described in Materials and Methods. 10 µg of nuclear extract was loaded into 10% gels. Gel electrophoresis and immunoblots were performed as described in Materials and Methods. Densitometry of bands was performed using Scion Image software Alpha 4.0.3.2. Protein expression of p65 was normalized to total protein and compared to levels in untreated cells (vehicle only) (* = 0.05). Values represent means +/− std error relative to vehicle control from 3 independent experiments. (B) Immunofluorescence of p65. Cells were treated as above. Immunoflourescence was performed as described in Materials and Methods. Top panel: vehicle; Middle pane: 40 µM CLA; Bottom panel: 80 µM CLA. Images were obtained using Delta Vision deconvolution microscope with SoftwoRX 3.5.0 software at 40× and optimized using Adobe PhotoShop CS2 version 9.0.2. (C) Western blot of total and phosphorylated IkappaB protein. Cells were plated and treated as above. 25 µg of whole cell lysate were loaded into 10% gels. Expression levels of IκB proteins were normalized to beta actin and compared to levels in untreated cells (vehicle only). Values represent means +/− std dev from 3 independent experiments (* = 0.05). Gel electrophoresis and immunoblots were performed as described in Materials and Methods. Densitometry of bands was performed using Scion Image software Alpha 4.0.3.2.

HER2 overexpression has been associated with an increase in NF-κB activity [9]. Pianetti et al., demonstrated that suppression of HER2 decreased NF-κB activity [42]. Based on this association and the observed suppression of HER2 protein by *t10c12* CLA, we tested whether nuclear localization of NF-κB was inhibited by *t10c12* CLA treatment. Nuclear extracts from SKBr3 cells treated with 40 and 80 µM *t10c12* CLA were isolated and examined for the relative expression of the NF-κB p65 subunit by Western blot and immunofluoresence. Figure 2A shows a representative Western blot of p65 protein expression following 24-hour treatment with *t10c12* CLA. Nuclear p65 was reduced at both the 40 and 80 µM doses in replicate experiments. The effect was statistically significant at 80 µM (p = 0.05) when compared to untreated controls. Immunofluorescence of anti-p65 in similarly treated cells confirmed an overall decrease in the p65 protein with CLA treatment (see Figure 2B).

Under basal conditions, NF-κB is sequestered in the cytoplasm by the IκB proteins. Phosphorylation of IκB by IKK targets the IκB complex for ubiquitination and proteosomal degradation, freeing NF-κB for nuclear localization [43]. To lend support to the data above, total and phosphorylated IκB-α protein levels were measured in whole cell lysates following similar treatments. In agreement with a downregulation of NF-κB, we observed a decrease in phosphorylated IκB protein levels at both the 40 and 80 µM dose, that was statistically significant, Figure 2C.

### Treatment with *t10c12* CLA or Celecoxib® Results in Decrease in PGE_2_ Levels and Suppression of HER2 Protein

**Figure 3:**
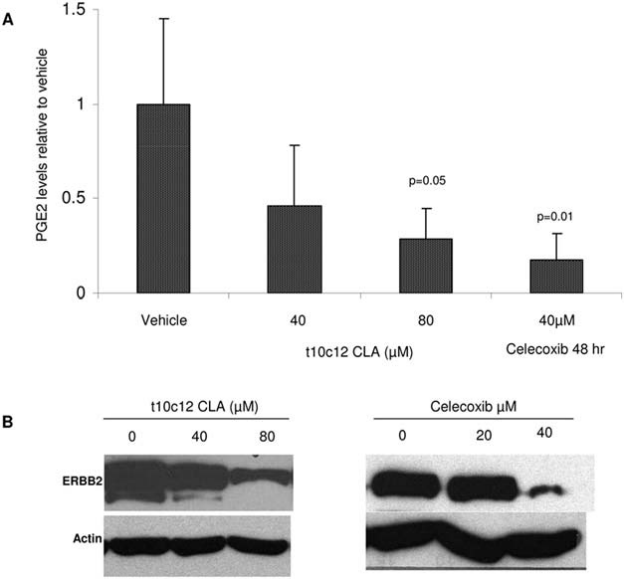
Suppression of HER2 and COX2 in SKBr3 cells. Cells were treated with *t10c12* CLA for 24 hrs or Celecoxib for 48 hrs. Cells were plated in 6-well plates, 3 wells per treatment. 30 minutes before collection, 15 µM arachidonic acid was added as substrate for COX2. Negative controls did not receive arachadonic acid. Cells from 3 wells were pulled for isolation of total protein and PGE_2_ determination by ELISA. (A) Western blots of HER2 protein following treatment with *t10c12* CLA or celecoxib. Gel electrophoresis and immunoblots were performed as described in Materials and Methods. Densitometry of bands was performed using Scion Image software Alpha 4.0.3.2. (B) PGE2 levels were measured by ELISA and are presented as pg/ml relative to vehicle. Values represent the means +/− std dev. from 2 independent experiments. (* = 0.05, ** = 0.001). Experiments were performed as describe in Materials and Methods.

Cyclooxygenases (COX) 1 and 2 are the rate-limiting enzymes in the conversion of arachadonic to prostaglandins. Constitutive COX1 is ubiquitously expressed and active in normal cellular processes. COX2 is induced as a consequence of NF-κB activation in response to various stimuli including stress, growth factors, cytokines, and oncogenes [13], [28]. COX2-derived PGE_2_ has been implicated in a number of pathways involved in tumorigenesis [28] and it has been shown to be a target is of CLA in a number of cell types [44], [45], [46]. We next asked if the apparent loss of NF-κB activity supported by the Western blots would correlate to a downregulation of COX2 activity. Supernatant obtained from the above experiments was used to measure levels of secreted PGE_2_ by ELISA as described in the Materials and Methods. As indicated in Figure 3A, *t10c12* CLA significantly inhibited PGE_2_ at the 80 µM dose (p<0.05). As positive control for COX2 downregulation, cells were treated with the COX2-specific inhibitor Celecoxib® at 20 and 40 µM for 48 hours. 40 µM Celecoxib® significantly inhibited PGE_2_ production by more than 80% (p<0.001). Consistent with a prior report that PGE_2_ influences HER2 expression [29], we found that reduction of HER2 correlated to a reduction in PGE_2_ synthesis (Figure 3B).

### The *t10c12* CLA Enhances Anti-Growth Effects of TNF-α and Herceptin® in SKBr3 Cells

**Figure 4:**
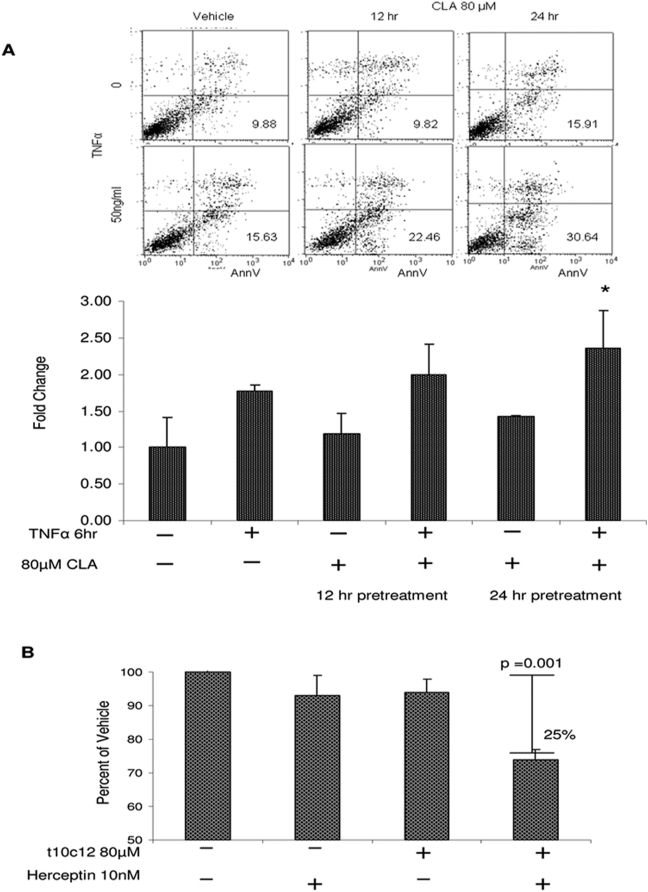
*t10c12* CLA enhances anti-growth effects of TNFα and Herceptin® in SK-Br3 cells. (A) CLA enhances TNF-α induced apoptosis. Cells were pretreated with 80 µM *t10c12* CLA for 12 or 24 hours before exposure to 50 ng/ml recombinant TNFα for 6 hours. Cells were plated in 6-well plates, 1 well per treatment. Annexin V staining was evaluated independently for each well and values were pulled to obtain an average Annexin V value per treatment condition. FACS plots are from a single experiment. Quantification of effect is presented as mean fold change +/− std dev in % Annexin V positive staining cells relative to vehicle control and were derived from 2 independent experiments. (p = 0.05). (B) Cells were pretreated with 80 µM *t10c12* CLA for 24 hrs, then co-treated with CLA +/− 10nM Herceptin® for an additional 24 hrs. Viability was assessed by MTT and absorbance at 580nm. Cells were plated in 96 well plates, 6 wells per treatment. Absorbance values were pulled for each treatment to obtain an average. Values represent the mean from 3 experiments +/− std. dev. The combination treatment significantly reduced absorbance compared to the vehicle control. Experiments were performed as described in Materials and Methods.

Overexpression of HER2 has been associated with attenuation of TNFα-induced apoptosis through upregulation of NF-κB [47], [48]. NF-κB inhibition has been shown to sensitize SKBr3 cells to TNFα [48], [49]. Based on the observed inhibition of NF-κB by the *t10c12* CLA, we predicted that pretreatment with the isomer would sensitize SKBr3 to TNFα. SKBr3 cells were pretreated with 80 µM *t10c12* CLA or vehicle control for 12 or 24 hours, media was removed, and cells were exposed to either TNFα in serum-depleted media (2% FBS) or serum-depleted media alone for an additional six hours. Both adherent and non-adherent cells were harvested and stained with FITC- Annexin V following the manufacturer's protocol (Sigma APOAF), and flow cytometry was performed to measure Annexin V positive cell fraction. As indicated in Figure 4A, exposure to 80 µM *t10c12* CLA showed a dose-dependent effect on the relative amount of Annexin V positive fraction following TNFα treatment that was statistically significant in 24-hour pretreatment conditions.

The monoclonal antibody trastuzumab (Herceptin®), which targets the extracellular domain of HER2, has been shown to inhibit proliferation in HER2-expressing cells, and inhibition of NF-κB has been shown to enhance these anti-proliferative effects in breast and colon cancer cells [29], [50]. Though SKBr3 cells have been demonstrated to be sensitive to Herceptin®, anti-proliferative response is not generally seen before three days of exposure and is enhanced in combination with other agents [51], [52], [53]. Based on its inhibition of NF-κB, we hypothesized that *t10c12* CLA would enhance the effect of Herceptin® in SKBr3 cells. In Figure 4B, cells were pretreated with 80 µM *t10c12* CLA for 24 hours, then co-treated with 10 nM Herceptin® for an additional 24 hours. The rationale for the pretreatment was based on the observed effect of 80 µM *t10c12* CLA on NF-κB downregulation in the SKBr3 cells. In preliminary dose escalation experiments, we did not detect a measureable effect of Herceptin® on proliferation in SKBr3 cells at any dose or exposure times tested (1–10 nM, 12–72 hours, data not shown). However, pretreatment with the *t10c12* CLA isomer significantly enhanced the anti-proliferative effects of Herceptin® by 25% (p = 0.001) compared to vehicle (Figure 4B). Neither Herceptin® nor CLA alone effectively inhibited proliferation at the doses and time points measured.

## Discussion

**Figure 5:**
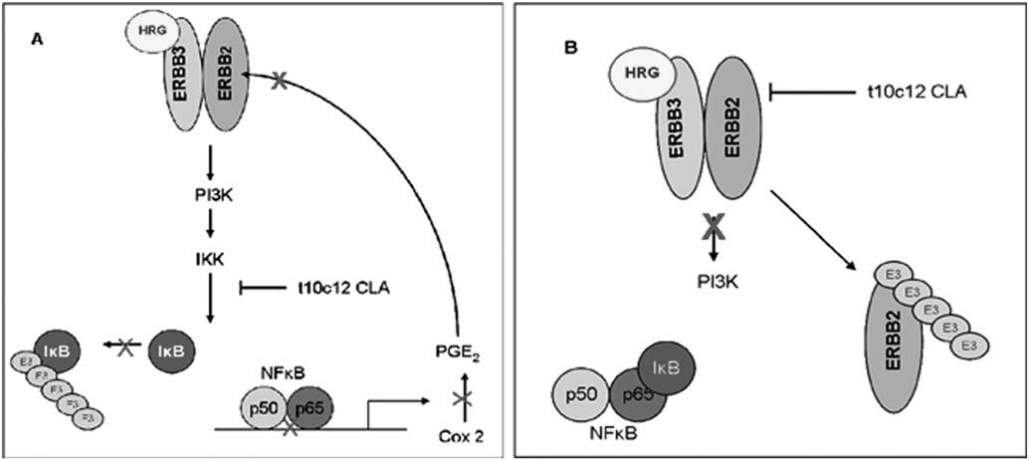
Proposed mechanism of CLA action in SKBr3 cells. (A) This scenario describes a direct effect on IKK by t10c12 CLA. A consequence of IKK inhibition is reduced phosphorylation of IκB and nuclear localization of p65. A decrease in COX2-derived PGE_2_ synthesis will result in loss of HER2 (as described in by Benoit et al, Oncogene, 2004; 23(8):1631). (B) This scenario describes a direct effect on HER2 protein by t10c12 CLA. In this scenario, HER2 is dissociated from the plasma membrane and targeted for ubiquitination and proteosomal degradation. Loss of HER2 signaling downregulates PI-3 kinase and IKK activity. NFκB is sequestered in the cyotosol by IκB and its target genes such as COX2 are not transcribed.

SKBr3 cancer cells are highly resistant to apoptosis [49], [54], a phenotype that is likely due to the overexpression of HER2 [52] and constitutive activity of the PI-3 kinase/AKT network, and NF-κB-induced signaling [6], [22], [42]. In this study, we show evidence that the *t10c12* isomer of CLA, at physiologically obtainable doses, significantly reduced HER2 protein in SKBr3 cells. Consistent with previous reports of NF-κB inhibition by CLA [36], [55], [56], 80 µM *t10c12* CLA significantly reduced NF-κB nuclear localization and the phosphorylation of IκBα. These data are noteworthy as they demonstrate the ability of the *t10c12* CLA isomer to target two key regulators in breast tumor promotion and treatment resistance, the HER2 receptor and NF-κB. These results support a mechanism of CLA that has not previously been demonstrated in an HER2-overexpressing breast cancer cell line. Although additional experiments are needed to confirm the direct target of CLA's action, we postulate two possible models for the observed effect of CLA on HER2 protein in SKBr3 cells illustrated in Figure 5.

In Figure 5A we suggest that CLA's inhibition of IKK indirectly downregulated HER2. As illustrated by the directional flow of the diagram, inhibition of IKK results in the downregulation of NF-κB and COX2-derived PGE_2_ levels. Based on published evidence supporting an inhibitor role of PGE_2_ on HER2 expression [29], a decrease in HER2 protein might also be observed by this mechanism. CLA has been associated with a decrease in IKK protein and activity in macrophages and attenuation of COX2 expression and PGE_2_ synthesis [36]. In addition to the protein analysis performed here, additional experiments, such as kinase activity assays for IKK α and β, are needed to confirm a direct action of CLA on IKK. Additionally, though not included here, an electrophoretic mobility shift assay (EMSA) would be informative in confirming the downregulation of NF-κB transcriptional activity that would be expected with a decrease in IKK activity.

Alternatively, the data presented here may be explained by Figure 5B where we suggest that the observed downregulation of NF-κB signaling is through a CLA effect on the membrane-bound HER2 protein. This may be through a direct action causing downregulation of protein expression, as has been demonstrated in the HT29 colon cancer cell line [38], dissociation of HER2 from its chaperone, HSP90, as has been demonstrated in a gastric cancer model [57], or disruption of caveoli or lipid rafts, which has been recently demonstrated in response to CLA [58]. Any of these scenarios could inhibit IKK through downregulation of the PI-3 kinase pathway, resulting in decreased nuclear p65 and COX2-induced PGE_2_ production, as is reported here.

Though not investigated here, CLA has been shown to downregulate COX2 by inhibiting 12-O-tetradecanoylphorbol-13-acetate (TPA)-induced AP-1 transcriptional activity [59]. As AP-1 proteins *c-fos* and *c-jun* have been shown to interact with nuclear p65 to enhance NF-κB promoter activity [60], an alternative scenario exists in which the downregulation of COX2 by *t10c12* CLA was through an AP-1 mediated mechanism. This possibility is supported by evidence in HER2 overexpressing breast epithelial cells in which overexpression of *c-jun* attenuated a pharmacological inhibition of AP-1 mediated COX2 activation [61].

There is a recognized need to develop non-toxic strategies to improve clinical outcome in HER2-positive breast cancers. Inhibition of NF-κB in combination with HER2-targeted therapies may enhance treatment response, as has been demonstrated with combination anti-COX2 and anti-HER2 therapies [50], [62], [63], [64]. The data presented here support further investigation into anti-NF-κB agents, such as specific isomers of CLA, in combination with Herceptin® in the treatment of a subset of breast cancers that are resistant to endocrine-based therapies.

There is a prevailing interest in the potential of bioactive compounds for their efficacy in tumor prevention and treatment. CLA has been demonstrated to have potent anti-tumor effects in some animal models of breast carcinogenesis [65]. Though poorly studied in humans for the prevention of breast cancer, studies in body composition have determined it to be non-toxic at doses up to 6g/day [66]. However, studies reporting adverse effects of supplementation with mixed-isomers or the *t10c12* isomer alone suggest caution, and this is emphasized by recent reports of tumor promoting activity of the *t10c12* isomer in animal models [67], [68]. Our data, however, add to a large body of work supporting an anti-tumor effect of the *t10c12* CLA isomer and warrant further investigation in the prevention and treatment of breast tumors overexpressing HER2/neu and NF-κB.
